# Investigation of Photocatalytic PVDF Membranes Containing Inorganic Nanoparticles for Model Dairy Wastewater Treatment

**DOI:** 10.3390/membranes13070656

**Published:** 2023-07-10

**Authors:** Elias Jigar Sisay, Ákos Ferenc Fazekas, Tamás Gyulavári, Judit Kopniczky, Béla Hopp, Gábor Veréb, Zsuzsanna László

**Affiliations:** 1 Doctoral School of Environmental Sciences, University of Szeged, Rerrich B. tér 1, H-6720 Szeged, Hungary; eliasjig@gmail.com (E.J.S.);; 2 Department of Biosystem Engineering, Faculty of Engineering, University of Szeged, Moszkvai Blvd. 9, H-6725 Szeged, Hungary; 3 Department of Applied and Environmental Chemistry, Institute of Chemistry, University of Szeged, Rerrich Béla Sqr. 1, H-6720 Szeged, Hungary; 4 Department of Optics and Quantum Electronics, Institute of Physics, University of Szeged, Dóm Sqr. 9, H-6720 Szeged, Hungary

**Keywords:** photocatalytic membrane, ultrafiltration, PVDF, bismuth vanadate, carbon nanotube, titanium dioxide

## Abstract

Membrane separation processes are promising methods for wastewater treatment. Membrane fouling limits their wider use; however, this may be mitigated using photocatalytic composite materials for membrane preparation. This study aimed to investigate photocatalytic polyvinylidene fluoride (PVDF)-based nanocomposite membranes for treating model dairy wastewater containing bovine serum albumin (BSA). Membranes were fabricated via physical coating (with TiO_2_, and/or carbon nanotubes, and/or BiVO_4_) and blending (with TiO_2_). Another objective of this study was to compare membranes of identical compositions fabricated using different techniques, and to examine how various TiO_2_ concentrations affect the antifouling and cleaning performances of the blended membranes. Filtration experiments were performed using a dead-end cell. Filtration resistances, BSA rejection, and photocatalytic cleanability (characterized by flux recovery ratio (FRR)) were measured. The surface characteristics (SEM, EDX), roughness (measured by atomic force microscopy, AFM), wettability (contact angle measurements), and zeta potential of the membranes were also examined. Coated PVDF membranes showed higher hydrophilicity than the pristine PVDF membrane, as evidenced by a decreased contact angle, but the higher hydrophilicity did not result in higher fluxes, unlike the case of blended membranes. The increased surface roughness resulted in increased reversible fouling, but decreased BSA retention. Furthermore, the TiO_2_-coated membranes had a better flux recovery ratio (FRR, 97%) than the TiO_2_-blended membranes (35%). However, the TiO_2_-coated membrane had larger total filtration resistances and a lower water flux than the commercial pristine PVDF membrane and TiO_2_-blended membrane, which may be due to pore blockage or an additional coating layer formed by the nanoparticles. The BSA rejection of the TiO_2_-coated membrane was lower than that of the commercial pristine PVDF membrane. In contrast, the TiO_2_-blended membranes showed lower resistance than the pristine PVDF membrane, and exhibited better antifouling performance, superior flux, and comparable BSA rejection. Increasing the TiO_2_ content of the TiO_2_-blended membranes (from 1 to 2.5%) resulted in increased antifouling and comparable BSA rejection (more than 95%). However, the effect of TiO_2_ concentration on flux recovery was negligible.

## 1. Introduction

Recently, membrane separation processes have gained attention in water and wastewater treatment applications. This is due to their efficient removal of pollutants, low environmental footprint, low cost, easy operation, and integrability with other processes [1,2]. Membrane separation processes applying ultrafiltration (UF) membranes have received substantial interest for efficiently removing pollutants from industrial wastewater [3,4]. Among industrial wastewaters, dairy wastewater generally contains proteins (besides fats and carbohydrates) that may cause severe membrane fouling, increasing the cost of the treatment. Polymeric membranes such as cellulose-based (CA) membranes and thin-film composite (TFC) membranes are commonly used in the industry for water purification [5]. Polyvinylidene fluoride (PVDF) is also a broadly applied polymeric material that has excellent mechanical strength, chemical resistance, oxidation resistance, and thermal stability [6,7,8]. PVDF membranes are susceptible to fouling by natural organic matter, proteins, and oily wastewater due to their less hydrophilic nature, resulting in reduced flux, reduced membrane shelf life, and increased energy costs [9]. Developing nanocomposite membranes by immobilizing nanomaterials in membranes or on membrane surfaces offers several benefits. These include photocatalytic oxidation of pollutants [10], antimicrobial effects [11], excellent antifouling properties, superior permeate quality, and long membrane lifetime [12]. It also provides the opportunity to use light to degrade pollutants in wastewater into harmless products such as carbon dioxide, ammonia, and water [13]. Membrane modification with titanium dioxide (TiO_2_) nanoparticles changes the chemical nature and pore size of membranes, and tends to reduce fouling in dairy protein separation processes [14]. 

Chemicals used for membrane cleaning may damage the membrane, generate effluents, and increase costs [14]. Many researchers have reported that TiO_2_-based nanocomposite membranes are suitable for photodegrading organic pollutants [13], which may cause a new membrane-cleaning method to emerge via the photodegradation of foulants. In addition to UV-active photocatalysts, several studies have recently been published on developing visible-light-active photocatalytic composite membranes. In recent years, bismuth-based oxides have gained attention in photocatalytic technology because of their narrow band gap and high visible light absorption; however, their relatively low specific surface area and activity hinder their broader application [15,16,17]. Combining carbon nanotubes (CNTs) with photocatalytic nanoparticles not only improves the hydrophilicity and antifouling property of membranes, but may also enhance photocatalytic efficiency by reducing the recombination rate of electron–hole pairs [13,18,19]. 

Several methods are applied for preparing nanoparticle-based membranes, including dip-coating, electrospinning, electro spraying, phase inversion, atomic layer deposition (ALD), and physical deposition [14]. Inorganic nanoparticle-based membranes can be prepared using phase inversion [12,20,21,22,23] or physical deposition methods [24,25], which are simple and effective ways to prepare photocatalytic membranes. However, to the best of our knowledge, a comparison of photocatalytic membrane performance for membranes fabricated by different methods and used for dairy wastewater treatment has not yet been investigated. 

Sisay et al. (2022) showed that considerable photocatalytic benefits can be gained using PVDF membranes blended with inorganic nanoparticles (TiO_2_–CNT–BiVO_4_) prepared via a phase inversion method [22]. However, there are no data published about the performance of TiO_2_–CNT–BiVO_4_-based composite membranes fabricated by physical deposition methods. Therefore, this study aims to prepare inorganic nanoparticle-containing membranes via physical coating methods, and investigate their antifouling and photocatalytic performance. Nanoparticle loading is a key parameter for membrane performance. Earlier studies showed that adding a small amount of TiO_2_ (up to 1.5%) to the dope solution resulted in greater porosity and pore size. However, further increasing the nanoparticle ratio in the membrane matrix (above 2%) decreased membrane performance: the best water flux and flux recovery ratio were achieved at a 1 wt% nanoparticle ratio [12]. Therefore, in this research, we applied a 1 wt% nanoparticle load. We prepared TiO_2_-modified PVDF membranes using blending and coating methods. The effects of the fabrication method and TiO_2_ concentration on the properties of the membranes (antifouling and membrane regeneration) during bovine serum albumin (BSA)-containing model dairy wastewater treatment were investigated.

## 2. Materials and Methods

In this study, both commercial and fabricated PVDF membranes were used. The commercial 30 kDa PVDF membrane (JW, GE Osmonics) was purchased from New Logic Research Inc., Minden, NV, USA. Non-commercial membranes were fabricated using PVDF powder (polymer), N-methyl-2-pyrrolidone (NMP) solvent, and sodium dodecylsulphate (SDS) surfactant. These materials were purchased from Merck Hungary (Budapest, Hungary). Membrane modification was carried out using multiwalled carbon nanotubes (CNTs; Kanto Chemical Co., Inc., Tokyo, Japan; TNMH3 15090, Japan; >98 wt%), Aeroxide P25 TiO_2_ (Merck EMD Millipore Co., Darmstadt, Germany), and bismuth vanadate (BiVO_4_). Bismuth vanadate was synthesized using bismuth(III) nitrate pentahydrate (Bi(NO_3_)_3_·5H_2_O; Alfa Aesar, ≥98%, ACS), nitric acid (HNO_3_; Merck, 99%), sodium hydroxide (NaOH) (Sigma-Aldrich Kft, Budapest, Hungary 100%, puriss), and ammonium vanadate (NH_4_VO_3_; Sigma Aldrich, ≥98%). Bovine serum albumin (69 kDa, ICN Biomedicals Inc., San Jose, CA, USA) was used as a model protein.

BiVO_4_ nanoparticles, referred to as “BiVO_4_-I sample” in the earlier study of Nascimben et al. (2020), were synthesized with a hydrothermal method. These BiVO4 nanoparticles proved stable, and had a narrow band gap of 2.35 eV [26]. XRD measurements proved that TiO_2_ and CNT are stable too [22].

Commercial PVDF membranes were used to prepare photocatalyst-coated membranes using a physical deposition method [27]. For this purpose, 0.04 g of commercial TiO_2_ was added to 100 mL of i-propanol and ultrasonicated for 3 min. Then, the ultrasonicated suspension was allowed to pass through a membrane in a dead-end cell at 0.3 MPa and dried for 1 h at room temperature before use. The samples were named (Table 1) in accordance with their preparation method and composition: *C*-refers to “coated” membranes, *P* is the membrane material (PVDF), *T* is titanium dioxide, *C* is carbon nanotube, and *B* is bismuth vanadate. The numbers represent the weight ratios of each constituent of the given nanoparticle mixtures. The detailed loadings and ratios of TiO_2_ and CNT or BiVO_4_ are listed in Table 1.

Pristine and photocatalyst-blended membranes were prepared using a phase-inversion method using a TQC sheen automatic film applicator (AB4120 081, The Netherlands). The compositions used for the preparation of ions of the casting dope solutions are shown in Table 2. The weight of the photocatalysts to be used was chosen so that the concentrations of the photocatalysts are identical in the blended membranes, as in the coated membranes. Thus, a 1 wt% nanoparticle loading was applied in all cases, except for those where the effect of TiO_2_ was investigated. 

The PVDF powder and photocatalysts were first dried in an oven at 80 °C for 4 h. Next, the dried PVDF powder was mixed with ultrasonicated photocatalysts for 1 min in 20 mL of NMP solution, and stirred for 12 h at 50 °C. Then, the solution was aged for 12 h without stirring in the dark. The dope solution was then ultrasonicated for 30 min, in order to remove air bubbles. Afterward, the solution was spread onto a glass plate with a 400 μm thick casting blade and allowed to form a skin layer for 30 s. Then, the glass plate and membrane were immersed in a bath containing a 3 g/L sodium dodecyl sulphate (SDS) solution at 10 °C for 3 h. During this process, phase inversion occurred between the water and NMP, creating pores in the membrane. SDS was used to prevent pore blockage and clean the pores. Finally, the membrane was cut to the desired size for characterization and filtration experiments. 

**Figure 1:**
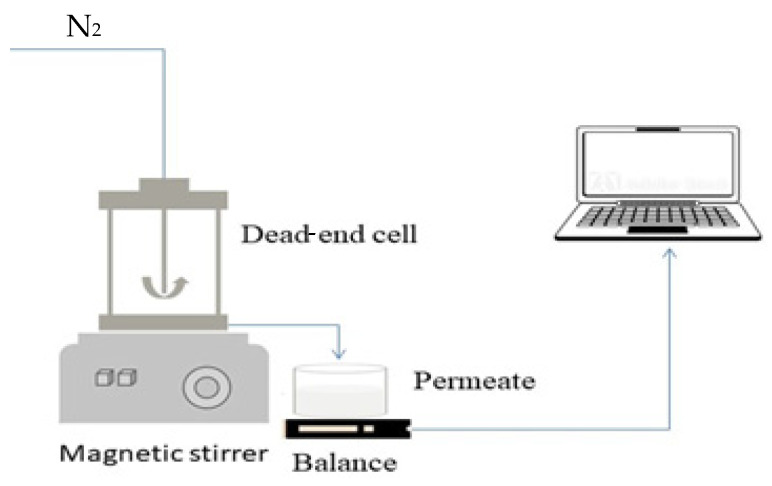
Graphical illustration of the filtration setup.

Filtration performance experiments were carried out with BSA-containing synthetic dairy wastewater, using a dead-end cell filtration setup (Millipore, XFUF04701, Merck KGaA, Darmstadt, Germany; Figure 1). As the main fouling components of dairy wastewater are proteins [28], a 1 g·L^−1^ BSA solution was applied to model dairy wastewater (this protein concentration is consistent with the protein content of real dairy wastewater [28]).

All of the membranes were pretreated with 250 mL of distilled water at 0.2 MPa before all measurements. Subsequently, the pure water flux and BSA rejection tests were performed at 0.1 Mpa. In each filtration, 250 mL of water or model solution was filtered until a volume reduction ratio (*VRR*) of five was reached. The *VRR* can be calculated using the following equation:

(1) $$VRR=\frac{VF}{VF-VP},$$

where *VF* and *VP* are the volumes of the feed and permeate (m^3^), at a given time, respectively.

The concentration of BSA in the model dairy wastewater was measured before and after filtration, using a spectrophotometric method. The measurement was based on recording the absorbance of BSA at a wavelength of 280 nm using a UV–visible spectrophotometer (Hitachi Co., U-2000, Tokyo, Japan). The samples were analyzed without further treatment. Chemical oxygen demand (COD) values of the samples were determined via the potassium–dichromate oxidation method. The samples (2 mL) were added to test tubes (Merck; in concentrations of 0–150 or 0–1500 mg·L^−1^) and digested for 2 h at 150 °C (Lovibond ET 108, Tintometer, Dortmund, Germany). Then, the absorbances were measured with a COD spectrophotometer (Lovibond PC-CheckIt, Tintometer, Germany). The turbidity of the samples was analyzed with a turbidity meter (Hach 2100AN, Berlin, Germany).

The surface roughness of the pristine and modified membranes was measured with a PSIA XE-100 atomic force microscope (AFM; South Korea; NC-AFM head mode) by evaluating the Rq values. At least three different 20 µm × 20 µm areas were scanned in each membrane; the obtained Rq values were averaged, and the standard deviations were calculated.

The surface of the membranes was characterized with a scanning electron microscope (SEM, Hitachi S-4700 Type II) using a 10 kV accelerating voltage. Elemental analysis was performed both for clean and fouled membranes with a Röntec X-Flash energy dispersive X-ray (EDX) detector (20 keV). To investigate the fouled membranes, we filtered 200 mL of 1 g/L BSA solution through the membrane, and then the membrane was dried without washing and used for measurements.

The hydrophilicity of the membranes was described by measuring contact angles using the sessile-drop method with a contact angle measuring instrument (OCA15Pro, Dataphysics, Filderstadt, Germany). For this purpose, 10 μL of ultrapure water was dripped from a micro-syringe onto the membrane surface. Then the image of the water droplet was recorded via a digital camera. An average of six parallel measurements was considered.

Streaming potential measurements were conducted to calculate zeta potentials using the Helmholtz–Smoluchowski (H–S) equation [29]. The measurements were carried out using SurPASS 3 adjustable gap cells. The surface zeta potential was determined in a 0.001 M potassium chloride (KCl) electrolyte solution in the 1–9 pH range. The H–S equation is (1) [29] as follows:

(2) $$\zeta =\frac{\Delta E}{\Delta P}\cdot \frac{\eta }{\varepsilon_{rel}\cdot \varepsilon_{0}}\cdot \;K_{B},$$

where *ζ* is the apparent zeta potential (mV), $\frac{\Delta E}{\Delta P}$ is the streaming potential developed as a result of an applied pressure gradient (V), *η* is the dynamic viscosity (Pa·s) of water, *ℇ_rel_* is the dielectric coefficient (–) of water, *ℇ*_0_ is the permittivity of vacuum (Fm^−1^), and *K_B_* is the electric conductivity of the aqueous solution (S·m^−1^). 

The fluxes were obtained using Equation (3):

(3) $$J=\frac{V}{A\cdot t}$$

where *J* refers to the flux (L·m^−2^·h^−1^), *W* refers to the volume of permeate (L), *A* means the effective surface area of the membrane (m^2^), and *t* means time (h). 

To evaluate the antifouling properties of all of the fabricated membranes, we subjected all membranes to 30 min of filtration with distilled water. A pressure of 0.2 MPa was used to ensure membrane compaction. Subsequently, the pure water flux (*J*_0_) filtration experiment was performed at 0.1 MPa. Then, the BSA solution (1 g·L^−1^), used as a foulant, was filtered through a dead-end filtration cell, and its permeation flux (*J_w_*) was measured. Afterward, the used membranes were washed with distilled water and reused to measure the second pure water flux (*J_w_*). 

Table 3 illustrates the resistances-in-series model, which includes membrane resistance (*RM*) and fouling resistances, that is, reversible (*Rrev*) and irreversible resistances (*Rirr*), that may arise during filtration. To determine the total resistance (*RT*), we calculated the sum of the membrane resistance, reversible resistance, and irreversible resistance. The fluxes were measured for the clean membranes, for the fouled membranes obtained after wastewater filtration, and for the cleaned membranes obtained after rinsing with distilled water using Equations (4)–(7) (Table 3) [30]. 

The antifouling performance was determined by carrying out the same experiments using the flux recovery ratio (FRR) Equation (8):

(8) $$\mathrm{FRR}=\frac{J_{c}}{J_{0}}\cdot 100\%\;,$$

where *J*_0_ is the water flux of the clean membrane (L·m^−2^·h^−1^), and *J_c_* is the water flux of the used membrane after cleaning (L·m^−2^·h^−1^). 

The membrane regeneration efficiency was examined by performing flux recovery experiments in the photocatalytic membrane reactors. After carrying out the filtration of the BSA solution and the flushing of membranes with distilled water, we measured the water fluxes. The fluxes were remeasured after 3 h and 21 h of UV (360 nm; in the case of TiO_2_ and/or CNT-containing composites) or visible light (“cool white”; in the case of BiVO_4_-containing composites) irradiation to describe the efficiency of photocatalytic flux recovery. The rejection of protein was calculated using Equation (9):

(9) $$\mathrm{Rejection}\left(\%\right)=\frac{c_{1}-c_{2}}{c_{1}}\cdot 100\%\;,$$

where *c*_1_ is the concentration of feed, and *c*_2_ is the concentration of the permeate. 

For statistical analysis, the Microsoft Excel software (version 2306) was used. At least three parallel measurements were performed in all cases, and the standard deviations were also considered.

## 3. Results

### 3.1. Investigation of the Stability of Nanoparticle-Coated Membranes

PVDF membranes coated with TiO_2_ were prepared with physical deposition and evaluated in terms of membrane stability via the mass retention ratio (MR), flux retention ratio (FR), and turbidity measurements. The MR and FR were 98.13% and 99.14%, respectively. Additionally, the turbidities of suspensions containing 5, 10, 20, and 40 mg of TiO_2_ were compared (Table 4). The turbidities of the solution-coated membranes were found to be insignificant compared to the turbidity values of the solutions containing 5 mg, 10 mg, and 20 mg of TiO_2_. Therefore, the results suggest that TiO_2_ may have leached slightly from the coated membranes.

### 3.2. Characterization of Nanoparticle-Coated Membranes 

#### 3.2.1. Surface Roughness

**Figure 2:**
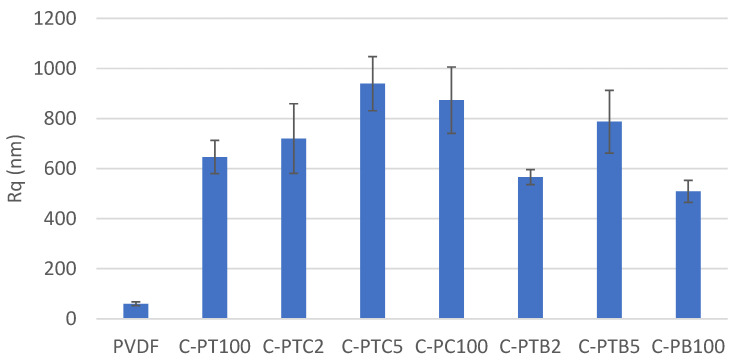
Surface roughness of coated membranes, where C-PTC means PVDF membranes coated with TiO_2_ and different concentrations of CNTs, while C-PTB means PVDF membranes coated with TiO_2_ and various amounts of BiVO_4_.

**Figure 3:**
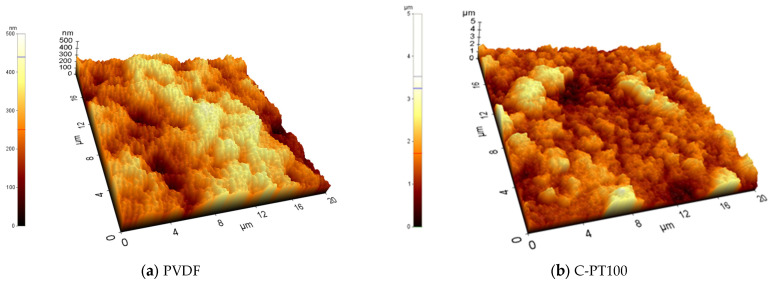

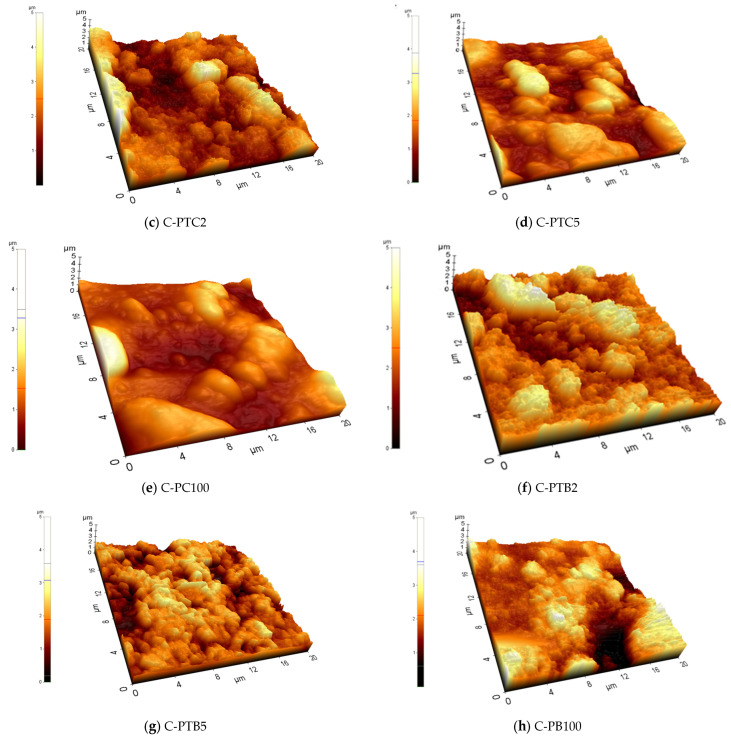
AFM pictures of coated membranes, where C-PTC means PVDF membranes coated with TiO_2_ and different concentrations of CNTs, while C-PTB means PVDF membranes coated with TiO_2_ and various amounts of BiVO_4_.

Eight membranes were selected (PVDF, C-PT100, C-PTC2, C-PTC5, C-PC100, C-PTB2, C-PTB5, and C-PB100), and their morphology and roughness were studied. The AFM measurements revealed that the covered membranes had a much higher surface roughness than the pristine PVDF membranes (Figure 2 and Figure 3).

**Figure 4:**
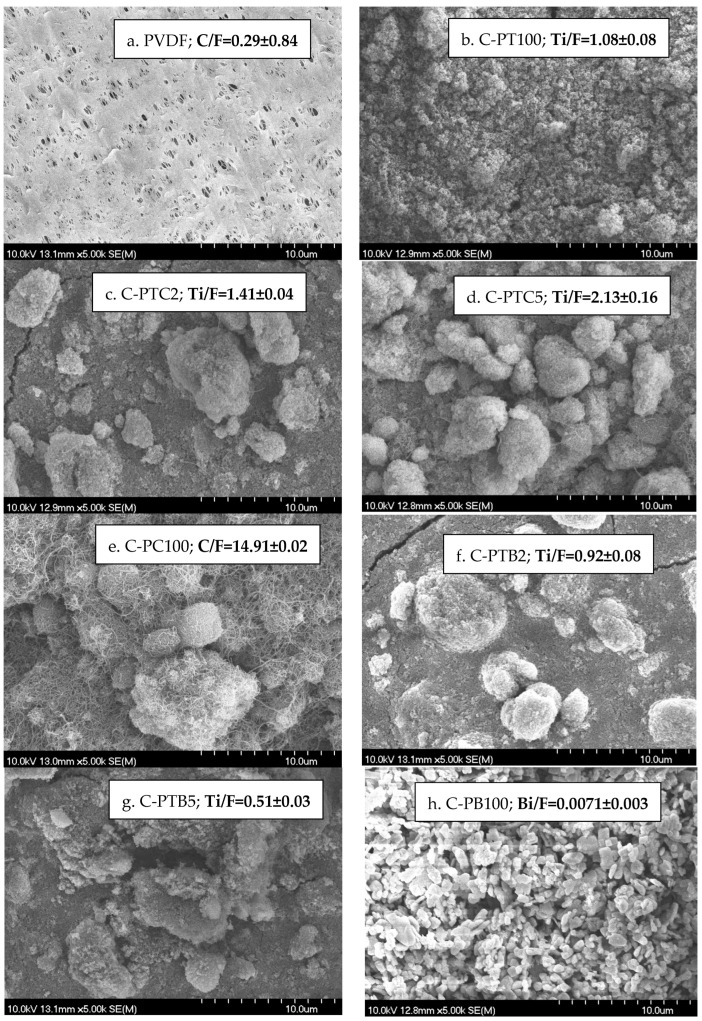
SEM images of clean membranes and NP/F ratio based on EDX analysis.

The SEM (Figure 4) and EDX results (Supplementary Material, Figure S1) revealed that the nanoparticles were aggregated and did not cover the surface evenly. In the TiO_2_-containing membranes, the Ti/F ratio shows the uniformity of coverage; these results show that the increasing CNT content resulted in a higher coverage, as the increasing Ti/F ratio demonstrates. An opposite effect can be observed in the case of increasing the BiVO_4_ content, which resulted in a less uniform coverage (Figure 4).

#### 3.2.2. Water Contact Angle Measurements

**Figure 5:**
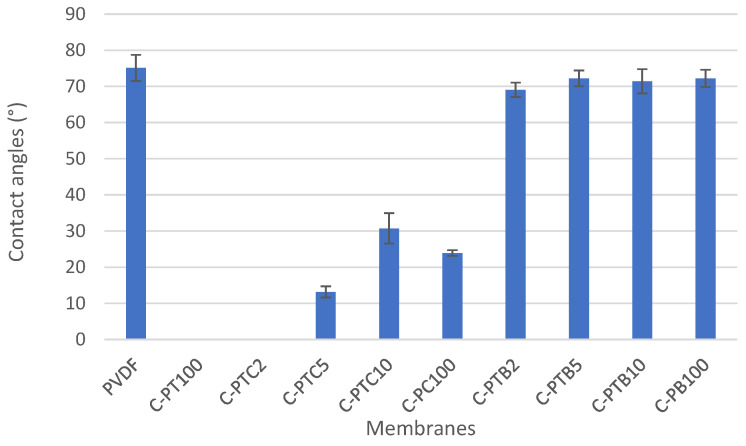
Contact angles of coated membranes, where C-PTC means PVDF membranes coated with TiO_2_ and various amounts of CNTs.

To evaluate the hydrophilicity of the prepared membranes, we measured the water contact angles at six sample points, and the mean ± standard deviation values are presented in Figure 5. The contact angle of the pristine PVDF membrane was 75.1 ± 3.63, indicating a slightly hydrophilic nature. It was observed that the addition of TiO_2_ significantly increased the hydrophilicity of the PVDF membrane, as evidenced by a contact angle of 0°, which means that the droplet completely spreads on the surface. This improvement in hydrophilicity positively impacts the water permeability of the membranes. Similarly, the addition of CNT decreased the contact angle, and the membrane coated with only CNT (C-PC100) exhibited high hydrophilicity. The effect of CNT may be related to the formation of bonds between the open ends of CNTs and the PVDF, increasing the negative charge density on the surface [31]. However, a better result was obtained after adding the TiO_2_–CNT mixture with 2% CNT content. Further increasing the CNT ratio resulted in decreased hydrophilicity, probably due to its more hydrophobic nature compared to TiO_2_. A similar study [12] reported hydrophilicity improvements for pristine PVDF membranes modified with nanoparticles. On the other hand, compared to the pristine PVDF, the addition of BiVO_4_ only slightly increased the hydrophilicity, even in the presence of TiO_2_. Nevertheless, surface hydrophilicity is jointly determined by the chemical composition of the surface, the surface roughness, and the extent of covering [32,33] (Figure 4).

#### 3.2.3. Zeta Potential of TiO_2_-Coated PVDF Membranes

**Figure 6:**
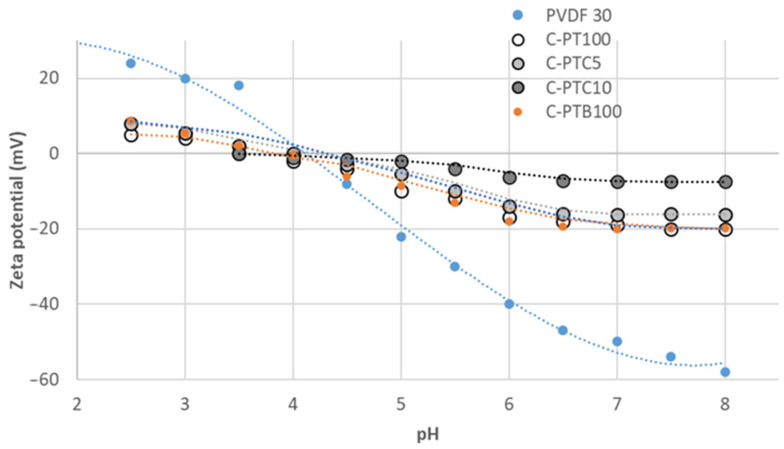
Dependence of zeta potential as a function of pH for PVDF and TiO_2_ and/or CNT or BiVO_4_ coated membranes.

As indicated in Figure 6, zeta potentials decrease with increasing pH values. The isoelectric points of the pristine and TiO_2_-coated (C-PT) membranes were observed at pH 4 and 3.6, respectively. The zeta potential of both membranes was negative at a neutral pH between −50 mV and −19 mV. The zeta potential results indicate that the surface charges of the pristine membranes were changed due to the modification; the PVDF-TiO_2_ and PVDF-BiVO_4_ membranes showed similar changes (their zeta potential was less negative). The addition of carbon nanotubes did not affect zeta potentials below pH 5, while at higher pHs, the zeta potentials increased with CNT concentration: at a neutral pH, they increased from −16 to −7.5 mV with the increase in CNT content from 5% to 10%. Modifying the membranes decreased their zeta potentials, caused by a shift in their surface electric charges towards neutrality, originating from an approximately equal amount of positive and negative charges. According to the antifouling theory [34], hydrophilic and electroneutral modified membranes are likely to have favorable antifouling performance.

### 3.3. Filtration Performance of Pristine and Physically Coated PVDF Membranes

This study aimed to examine the impact of nanoparticle coatings on the filtration performance of PVDF membranes. For this purpose, we prepared and compared physically coated membranes (C-PVDF–TiO_2_, C-PVDF–TiO_2_–CNT, and C-PVDF–TiO_2_–BiVO_4_).

#### 3.3.1. Effects of Nanoparticle Coating on Flux and Filtration Resistances during the Filtration of Model Wastewaters

**Figure 7:**
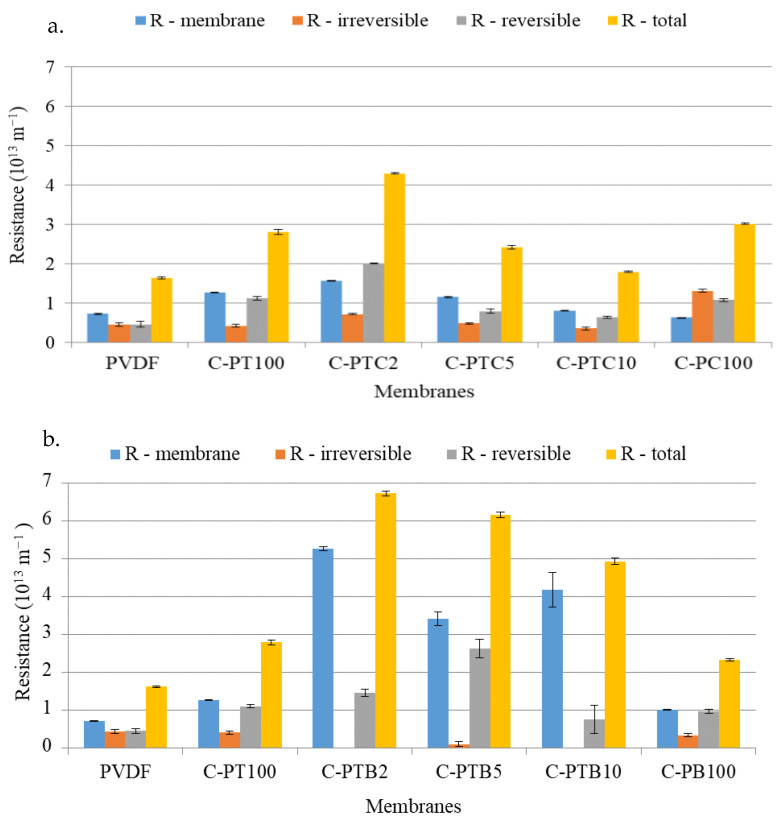
Filtration resistances of TiO_2_–CNT-coated PVDF membranes (**a**) and TiO_2_–BiVO_4_-coated PVDF membranes (**b**), where C-PTC means PVDF membranes coated by TiO_2_ and different concentrations of CNTs, and C-PTB means PVDF membranes coated with TiO_2_ and different concentrations of BiVO_4_.

The impact of nanoparticles on filtration efficiency was investigated using the resistances-in-series model. The filtration resistances were calculated based on the fluxes observed during the filtration of BSA-containing model wastewater. *RM*, *Rirr*, *Rrev*, and *RT* values were calculated using Equations (4)–(7). Coating with TiO_2_ and CNT significantly increased the total filtration resistances (Figure 7a), as evidenced by the increased reversible resistances (which can be easily eliminated by rinsing the membrane surface). Our observations align with those of previous studies [34,35] that showed that adding more nanoparticles to the membrane surface resulted in pore blockage and reduced water flux. An analysis of variance (ANOVA) was also carried out to evaluate the results further. Regression results show no significant difference between the results obtained during repeated measurements. However, there is a significant difference in the total and irreversible resistances of the pristine PVDF membrane (control) and modified membranes. 

#### 3.3.2. Effects of Nanoparticle Coating on the Rejection of BSA

**Figure 8:**
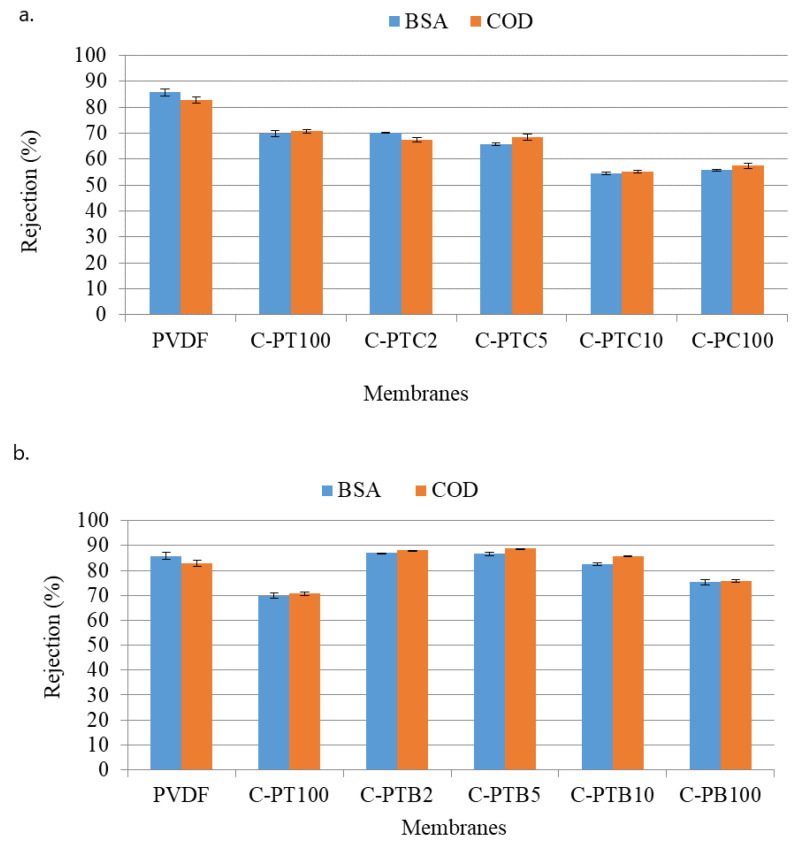
BSA and COD rejection performances of TiO_2_–CNT-coated PVDF membranes (**a**) and TiO_2_–BiVO_4_-coated PVDF membranes (**b**).

BSA and COD rejections of TiO_2_–CNT-coated PVDF membranes and TiO_2_–BiVO_4_-coated PVDF membranes were investigated and compared (Figure 8). The former (Figure 8a) showed a decreasing trend of BSA and COD rejection performances as the CNT content increased. The latter (Figure 8b) exhibited similar BSA and COD rejection performances as the pristine PVDF membrane. This could be due to the strong repulsion force between the membrane surface and BSA molecules at higher pHs.

**Figure 9:**
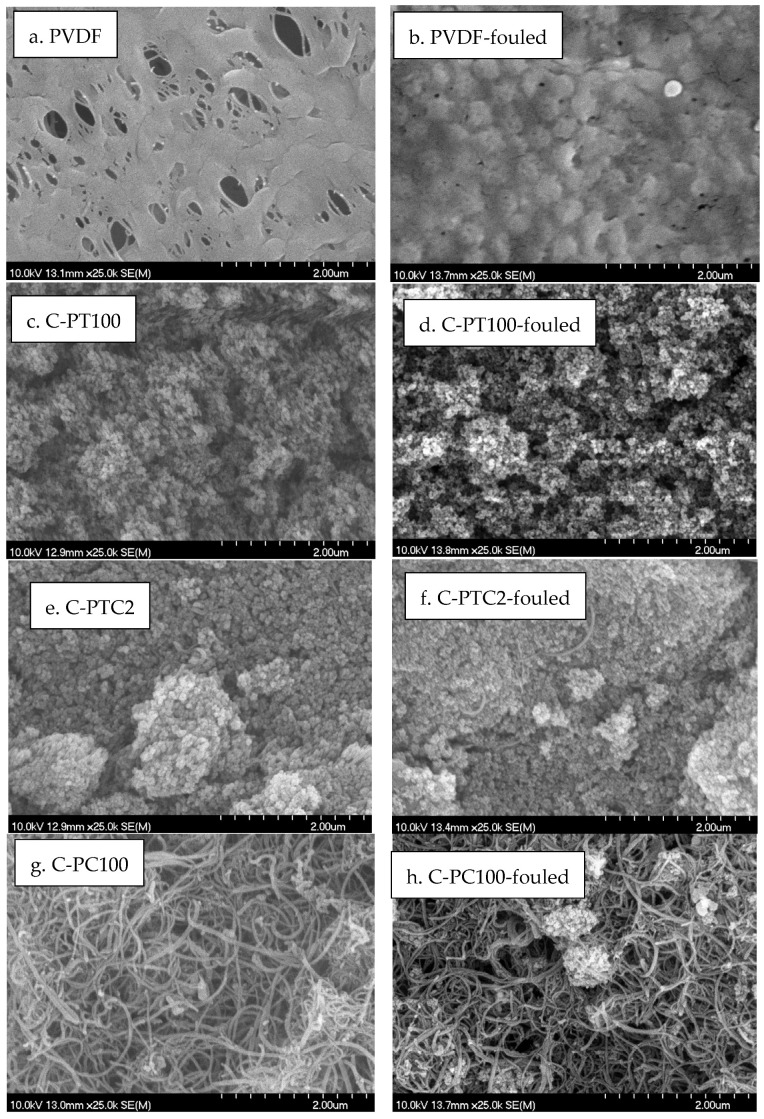

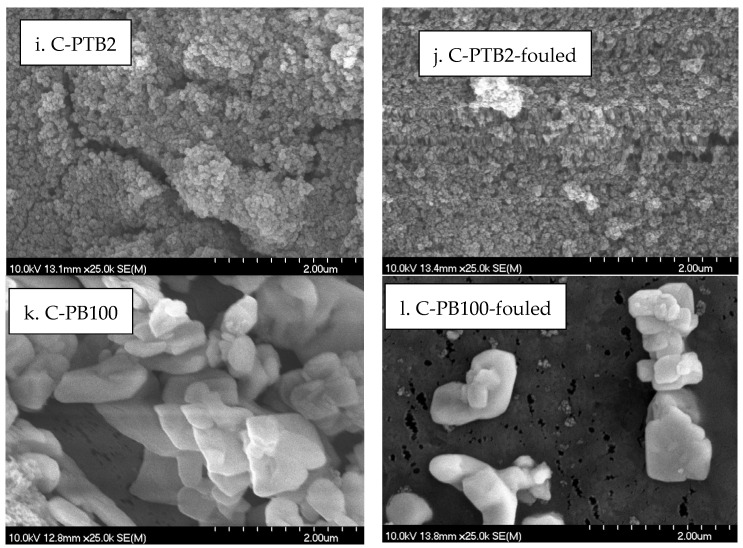
SEM micrographs of clean and BSA-fouled membranes’ surfaces.

**Figure 10:**
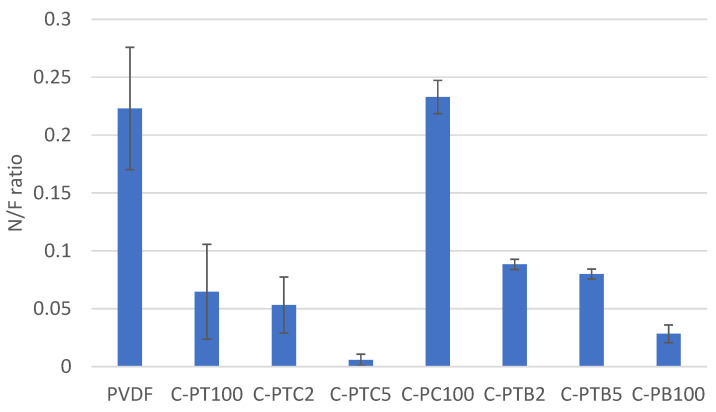
N/F ratios for the surfaces of fouled membranes.

The unmodified PVDF membrane demonstrated the best rejection rates for BSA (86%) and COD (83%) compared to all of the other, modified membranes. This result is surprising, as the modification resulted in increased filtration resistances; thus, increased protein rejection was expected for the modified membranes. Nevertheless, this finding is in accordance with the results obtained by Farahani et al. [12]. To explain this behavior, we recorded SEM images of the fouled membranes. Moreover, the nitrogen/fluorine ratios were calculated based on EDX measurements (Supplementary Material, Figure S2.). Higher N/F ratios mean that BSA covers a higher surface area, as the nitrogen content originates from BSA, while fluorine content is related to the membrane material (PVDF). Although the NPs cover the membrane surface (Figure 4), the EDX and SEM results show that the coating is not compact, leaving the membrane surface available. A possible explanation for this unexpected result can be related to the structure of the surface: the nanoparticle-covered membrane surfaces are rougher (Figure 2), which prevents the development of an intact gel layer that may cover the pores and act as another filtration layer (Figure 9a,b). This interpretation is supported by the decreased N/F ratio of the fouled modified membranes (Figure 10). The only exception is the CNT-covered membrane, which can be explained by the relatively high coverage of the surface (Figure 4) and by the finding that BSA molecules accumulated in the larger fibers of the CNTs (Figure 9g,h). Moreover, the NP-containing surfaces may have altered the structure, behavior, and function of the proteins, allowing them to pass through the membrane with ease and reducing rejection rates, as demonstrated for C-PB100 (Figure 9k,l), where much smaller BSA aggregates can be observed compared to the other membranes. 

#### 3.3.3. Regeneration of Physically Modified Fouled Membranes

**Figure 11:**
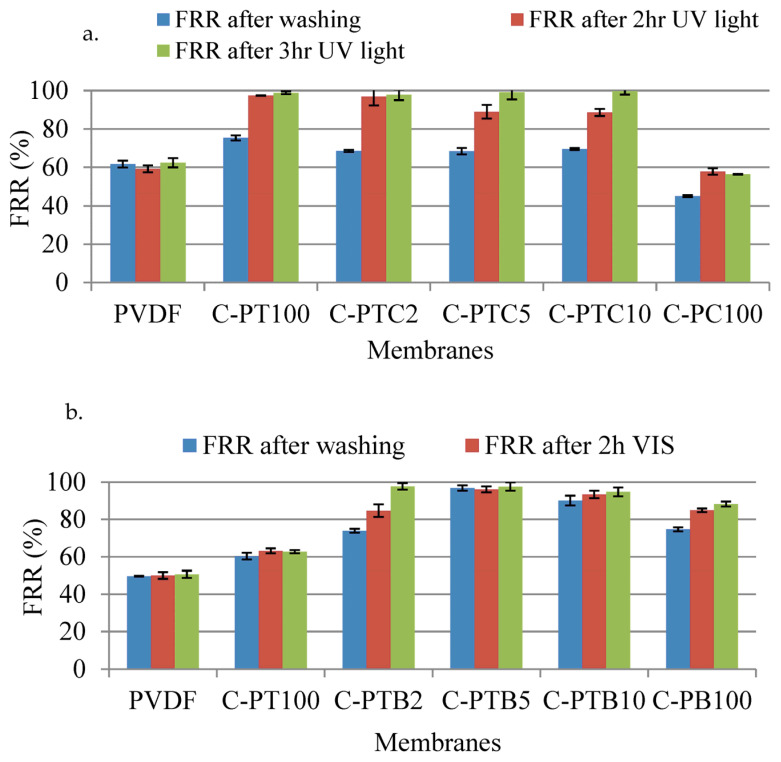
Regeneration of BSA-fouled TiO_2_–CNT-coated PVDF membranes under UV light irradiation (**a**) and PVDF-, PVDF–TiO_2_-, and TiO_2_–BiVO_4_-coated PVDF membranes under visible light irradiation (**b**).

Regeneration experiments were conducted on fouled membranes using UV light for TiO_2_ and TiO_2_–CNT-containing membranes (C-PT and C-PTC) or visible light for BiVO_4_-containing membranes (C-PB). The results show that all coated PT and PTC membranes achieved more than 97% recovery under UV light, whereas this value was only 60% for the pristine PVDF membrane (Figure 11). After 3 h of UV light exposure, coated C-PTC5 (99.13%) and PTC10 (99.70%) membranes showed slightly better regeneration than the C-PT100 (98.80%). The reason for this phenomenon may be attributed to reduced electron–hole recombination, and the enhancement of the photocatalytic activity of TiO_2_ due to the presence of CNT acting as an electron–hole sink [35]. After 2 h of UV light exposure, the best regeneration was obtained by C-PTC2. 

The coated PC100 membrane showed the lowest regeneration ratio. In contrast, all of the TiO_2_–BiVO_4_-coated PVDF membranes and C-PB membranes showed better regeneration ratios under visible light compared to the pristine PVDF or C-PT membranes. The FRRs of coated C-PTB2 and C-PB100 after 2 h of visible light exposure were approximately 84.7% and 84.90%, respectively. However, the other TiO_2_–BiVO_4_-coated PVDF membranes did not exhibit any regeneration due to the absence or low amount of irreversible foulants after washing with water (Figure 11). The regression results of the pristine (control) and modified membranes for the flux recovery ratio were significant according to ANOVA calculations. This indicates that the photocatalytic property of the modified membranes was significantly improved.

### 3.4. Photocatalytic Blended Ultrafiltration Membranes

Next, photocatalytic nanocomposite membranes were fabricated by incorporating inorganic nanoparticles into the membrane material during the phase inversion method. The experiment aimed to examine how the concentration of TiO_2_ affects the filtration and regeneration performances of PVDF membranes during filtration of the BSA solution. Thus, blended PVDF–TiO_2_ photocatalytic ultrafiltration membranes (referred to as B-PT membranes) with different TiO_2_ concentrations were prepared. Then, the filtration and regeneration performances of the pristine PVDF membrane and the PVDF–TiO_2_ photocatalytic blended membranes were compared.

#### 3.4.1. Contact Angle, Water Flux, and Rejection Performance of Blended Membranes

To evaluate the hydrophilicity of the membrane surface, we measured the contact angles of distilled water at the membrane surface. Table 5 shows the contact angles, water flux, and BSA rejection characteristics of B-PT ultrafiltration membranes at various concentrations of TiO_2_. As the concentration of TiO_2_ was increased from 0 to 2.5%, the contact angles of the B-PT membranes decreased, and the water flux increased. These findings show that increased TiO_2_ concentration resulted in increased hydrophilicity compared to pristine PVDF. This finding is similar to that observed for the coated membranes; however, the presence of PVDF membrane material on the surface prevents the development of superhydrophilic membrane surfaces (i.e., a contact angle of 0°), unlike in the case of coated membranes. Our results agree with those obtained by Farahani et al. [12]. The fabricated pristine PVDF membrane showed a BSA rejection of 98.88%, and a similar rejection performance was observed for the B-PT1 and B-PT1.5 membranes (Table 5).

#### 3.4.2. Filtration Resistances of Blended PVDF–TiO_2_ Photocatalytic UF Membranes

**Figure 12:**
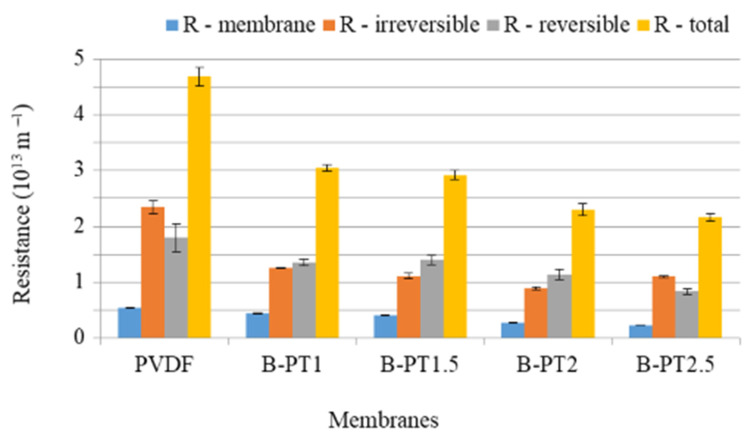
Filtration resistances of blended TiO_2_-containing PVDF membranes (B-PT) containing various amounts of TiO_2_.

To evaluate the efficacy of the modified membranes in fouling mitigation, we filtered the BSA-containing protein solutions and determined the filtration resistances using Equations (4)–(7). The resulting *RM*, *Rirr*, *Rrev*, and *RT* values are presented in Figure 12. The pristine membrane exhibited the highest filtration resistances and notably high irreversible fouling. However, modification of the membrane led to a significant reduction in filtration resistances, with the resistances decreasing further as the concentration of TiO_2_ increased (Figure 12).

In contrast to the neat membrane, the membranes containing 1, 1.5, and 2% TiO_2_ showed higher reversible fouling resistances than irreversible fouling. This could be explained by the increased hydrophilicity of the membrane surface (as proven by the decreased contact angles), preventing the formation of strong bonds between BSA and the membrane surface, which would render the membrane unwashable. These findings are consistent with those of previous studies [36,37]. However, at higher TiO_2_ concentrations, the trend was reversed, which could be attributed to pore blockage caused by nanoparticles or agglomerated NPs at higher concentrations. Consequently, this phenomenon may reduce the beneficial effects of hydrophilicity and morphology on water permeation [12].

#### 3.4.3. Regeneration of BSA-Fouled Blended PVDF–TiO_2_ Photocatalytic UF Membranes

The objective of the regeneration experiment was to investigate the impact of TiO_2_ concentration on the regeneration performance of BSA-fouled B-PT membranes under UV light. The experiment aimed to assess the photocatalytic degradation of the irreversible foulants attached to the membrane during the filtration of BSA solution at its own pH of 6.5 ± 0.08.

**Figure 13:**
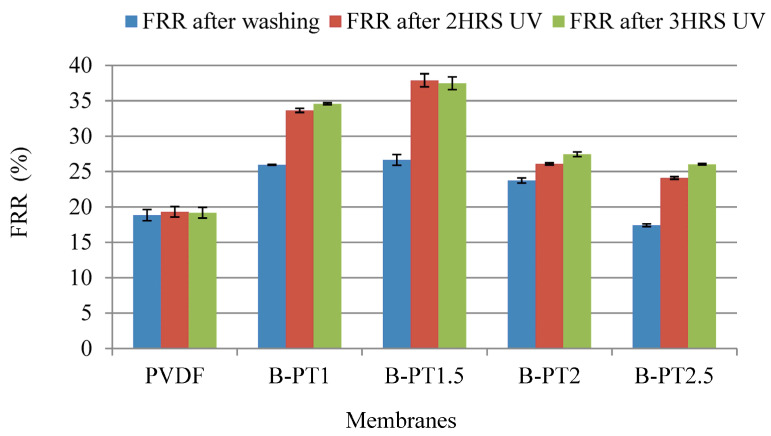
Regeneration of BSA-fouled PVDF and B-PT UF membranes containing various amounts of TiO_2_.

Figure 13 shows the regeneration of BSA-fouled B-PT photocatalytic UF membranes. The BSA-fouled pristine PVDF and B-PT photocatalytic membranes were cleaned using distilled water and UV radiation (*λ*_max_ = 360 nm) for 2 and 3 h. It was observed that the flux improved after cleaning the B-PT membranes using UV radiation, but the degree of flux recovery for all photocatalytic membranes was minor. The highest regeneration performance was observed for B-PT1.5 (FRR = 37.47%), while the lowest was obtained for B-PT2.5 (FRR = 26.02%) after 3 h of UV light exposure. The lower regeneration at higher TiO_2_ concentration may be due to the greater tendency of irreversible fouling in larger pores, as observed for B-PT2 and B-PT2.5.

## 4. Discussion

Our research revealed that coating PVDF membranes with TiO_2_ rendered them very hydrophilic (0° contact angle means that the water droplet completely spreads on the surface), which was expected to affect the water permeability of the membranes positively. The addition of CNT also decreased the contact angle: the membrane covered solely by CNT (C-PC100) had high hydrophilicity, even in the absence of TiO_2_. Controversially, the higher hydrophilicity did not result in higher flux due to the resistance of the coating layer itself. The highest resistance was observed for the C-PTB membranes, in which the TiO_2_ particles were aggregated and did not cover the membrane according to the low nanoparticle/F ratio of the surface. The increased membrane resistances show that the nanoparticles foul the membrane pores.

Decreased BSA rejection was observed for the coated membranes compared to the pristine membrane. This can be explained by the formation of a gel layer on the surfaces of membranes. A more compact gel layer can form on the pristine PVDF membrane, serving as a filtration medium for BSA. The more uneven distribution of BSA on the surface of coated membranes allows BSA to pass through the membrane and reduce BSA rejection. The worst BSA rejection was observed for the C-PTC membranes, for which a relatively well-covered (proved by the NP/F ratio) but rough surface was observed, limiting the ability of BSA to adhere to the surface of the PVDF membrane. In the C-PTB membranes, although the aggregates resulted in a rough surface, the uneven coverage could not prevent the formation of a gel layer, resulting in higher BSA resistances.

In contrast, the BSA rejection of all blended membranes (97%) was comparable with that of the pristine PVDF membrane. These results are in accordance with recent experiments where 97–99% BSA rejections were achieved using TiO_2_-coated PES membranes, while the flux recovery ratios were 70–80% after the filtration of BSA [36]. More than 97% regeneration was achieved for our C-PT and coated C-PTC membranes after 3 h of UV light exposure. In terms of membrane regeneration by visible light, earlier studies showed that a maximum of 75% flux recovery can be achieved using visible-light-active Fe(III)-TiO_2_/PVDF composite membranes [38], while our results showed about a 95–97% flux recovery after the filtration of BSA. For blended membranes, opposite results were obtained, as the pristine membrane showed the highest filtration resistances with considerably high irreversible fouling. It was found that the modification significantly decreased the filtration resistances, probably because the inorganic nanoparticles in the blended membrane did not form an additional layer on the surface that could potentially block the pores and decrease the flux. The resistances also decreased with the increase in TiO_2_ concentration. In summary, in this study, membrane fouling was successfully reduced by incorporating inorganic nanoparticles. 

The flux also improved after cleaning the fouled blended-PT membranes under UV radiation; however, the flux recovery of all blended photocatalytic membranes was small compared to that of the coated membranes. Moreover, the effect of TiO_2_ concentration on the regeneration of the fouled B-PT membranes was negligible. Regeneration performances (expressed as FRR) after 3 h of UV light exposure varied between 37.47% and 26.02%. Better antifouling and more considerable BSA rejections could be obtained for membranes prepared with the phase inversion method compared to those prepared with the physical coating method. The flux recovery performance of the blended membranes was not as high as that of other blended membranes published earlier [22,39,40]. For the latter, 20% TiO_2_-containing PVDF membranes achieved 96.9% and 60.2% flux recoveries with and without UV regeneration [39], respectively. Thus, further research is required to improve the flux restoration of blended membranes. The performances of membranes prepared in this study were also compared with those prepared in other studies (Table 6). 

## 5. Conclusions

This research aimed to investigate the performance of PVDF-based nanocomposite membranes coated with TiO_2_, and/or CNT, and/or BiVO_4_ NPs for treating model dairy wastewater containing BSA. It was found that the composition of nanocomposites, together with properties such as aggregation, roughness, and the uniformity of covering, determine the fouling mechanism. Moreover, TiO_2_-containing blended PVDF membranes were prepared, and the effect of TiO_2_ concentration on their performance was also investigated. Two fabrication methods were applied and compared in terms of BSA filtration performance and regeneration performance of fouled membranes under UV or visible light. 

More than 97% regeneration was achieved for the C-PT and all C-PTC membranes under UV light. Moreover, all of the C-PTB membranes exhibited better regeneration under visible light than the pristine PVDF and PT membranes. After the ultrafiltration of the BSA model solution, the best flux recovery ratio was obtained for the C-PTC membrane (FRR = 96.8%) after 2 h of UV light irradiation. FRRs of 84% and 97.7% can be obtained for the C-PTB membranes after 3 h and 21 h of visible light irradiation, respectively. The flux restoration of all blended photocatalytic membranes was smaller than that of coated membranes, and the effect of TiO_2_ concentration on the regeneration of the fouled B-PT membranes was negligible. The highest regeneration performance (expressed as FRR) under 3 h of UV light exposure was 37.47%, while the lowest one was 26.02%. Better antifouling and more considerable BSA rejections can be obtained for membranes prepared with the phase inversion method than for membranes prepared with the physical coating method. However, further research is required to improve the flux restoration of blended membranes.

## Figures and Tables

**Table 1:** Loading and ratios of TiO_2_. CNT and BiVO_4_ nanoparticles (NPs) used for the preparation of coated membranes.

Membrane	TiO_2_ (g)	CNT (g)	BiVO_4_ (g)	Membrane Description
PVDF30	-	-	-	PVDF without nanoparticles
C-PT100	0.04	-	-	Coated (C) PVDF with 1% NP (100 wt% TiO_2_)
C-PTC2	0.0392	0.0008	-	C-PVDF with 1% NP (98 wt% TiO_2_ and 2 wt% CNT)
C-PTC5	0.038	0.002	-	C-PVDF with 1% NP (95 wt% TiO_2_ and 5 wt% CNT)
C-PTC10	0.036	0.004	-	C-PVDF with 1% NP (90 wt% TiO_2_ and 10 wt% CNT)
C-PTC100	-	0.04	-	C-PVDF with 1% NP (100 wt% CNT)
C-PTB2	0.0392	-	0.0008	C-PVDF with 1% NP (98 wt% TiO_2_ and 2 wt% BiVO_4_)
C-PTB5	0.038	-	0.002	C-PVDF with 1% NP (95 wt% TiO_2_ and 5 wt% BiVO_4_)
C-PTB10	0.036	-	0.004	C-PVDF with 1% NP (90 wt% TiO_2_ and 10 wt% BiVO_4_)
C-PB100	-	-	0.04	C-PVDF with 1% NP (100 wt% BiVO_4_)

**Table 2:** Composition of casting dope solutions used for membrane preparation.

Membrane	PVDF (g)	TiO_2_ (g)	Solvent (NMP, g)	Description
B-PDVF	3.5	0	16	Pristine PVDF
B-PT1	3.465	0.035	16	PVDF with 1% TiO_2_
B-PT1.5	3.4475	0.0525	16	PVDF with 1.5% TiO_2_
B-PT2	3.43	0.07	16	PVDF with 2% TiO_2_
B-PT2.5	3.4125	0.0875	16	PVDF with 2.5% TiO_2_

**Table 3:** Filtration resistances according to resistances-in-series model.

Filtration Resistances (m^−1^)	Formula	
Membrane resistance	$RM=\frac{\Delta P}{J_{0}\cdot \eta w}$	(4)
Irreversible resistance	$Rirrev=\frac{\Delta P}{J_{W}\cdot \eta w}-RM$	(5)
Reversible resistance	$Rrev=\frac{\Delta P}{J_{w}\cdot \eta ww}-RF-RM$	(6)
Total resistance	RT=RM+Rirr+Rrev	(7)

Δ*p* is the change of pressure (Pa), *J*_0_ is the water flux of the clean membrane, *J_w_* is the water flux of the fouled membrane, *J_W_* is the water flux obtained after rinsing the fouled membrane (L·m^−2^·h^−1^), η_W_ is the dynamic viscosity of water (Pa·s), and *η_ww_* is the dynamic viscosity of wastewater (Pa·s).

**Table 4:** Turbidity of distilled water containing a TiO_2_-coated membrane and various concentrations of TiO_2_.

No.	Composition (in 200 mL of Distilled Water)	Turbidity (NTU)
	Distilled water only	0.075 ± 0.00
1	Distilled water above TiO_2_-coated membrane (40 mg of TiO_2_)	1.55 ± 0.07
2	5 mg of TiO_2_	90.63 ± 0.27
3	10 mg of TiO_2_	190 ± 1
4	20 mg of TiO_2_	450.33 ± 2.89

**Table 5:** Characteristics of TiO_2_/PVDF photocatalytic blended ultrafiltration membranes.

Membrane Type	Water Contact Angle (°)	Water Flux (L·m^−2^·h^−1^)	Rejection (%)	
			BSA	COD
PVDF	78.1 ± 4.59 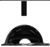	67.22 ± 0.7	98.88 ± 0.09	99.83 ± 0.08
B-PT1	73.45 ± 4.33 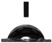	82.94 ± 1.56	97.59 ± 0.57	99.74 ± 0.09
B-PT1.5	72.26 ± 4.0.6 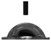	90.78 ± 1.33	99.06 ± 0.87	98.47 ± 0.09
B-PT2	70.48 ± 2.82 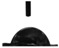	110.04 ± 1.30	97.74 ± 0.84	96.27 ± 0.04
B-PT2.5	66.72 ± 3.44 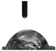	157.88 ± 1.41	95.8 ± 0.85	98.27 ± 0.26

**Table 6:** Comparison of membrane performances of PVDF membranes modified with 1% inorganic nanoparticles.

Membrane Type	Method of Preparation	Contact Angle (°)	Water Flux (L·m^−2^·h^−1^)	BSA rejection (%)	FRR (%)	
PVDF	blending	74.04 ± 1.5	50.96 ± 8.53	similar BSA retention	-	[40]
PVDF–TiO_2_	blending	63.09 ± 1.28	117.95 ± 8.96		-	[40]
PVDF–TiO_2_–BiVO_4_-50	blending	62.3 ± 4.24	153.56 ± 1	97.75 ± 0.03	59% (Vis)	[22]
PVDF–TiO_2_–CNT–BiVO_4_-50	blending	69.875 ± 5.01	150.52 ± 2.04	97.10 ± 0.77	50% (Vis)	[22]
PVDF	blending	78.1 ± 4.59	67.22 ± 0.7	98.88 ± 0.09	18% (UV)	this study
PVDF–TiO_2_	blending	73.45 ± 4.33	82.94 ± 1.56	97.59 ± 0.57	35% (UV)	this study
PVDF	Physical deposition	75.1 ± 3.63	50.73 ± 4.11	85.74 ± 0.055	60% (UV) 50% (Vis)	this study
PVDF–TiO_2_	Physical deposition	0.00 ± 0.00	28.8 ± 3.54	69.84 ± 0.76	97% (UV)	this study
PVDF–CNT	Physical deposition	23.92 ± 0.8	42.78 ± 4..01	55.75 ± 0.44	58% (UV)	this study
PVDF–BiVO_4_	Physical deposition	72.22 ± 2.37	35.89 ± 2.33	75.27 ± 0.69	90% (Vis)	this study

## Data Availability

Not applicable.

## Supplementary Materials

The following supporting information can be downloaded at: https://www.mdpi.com/article/10.3390/membranes13070656/s1, Figure S1: EDX spectra of covered nanocomposite membranes; Figure S2: EDX spectra of covered nanocomposite membranes after BSA filtration.

## References

[1] Chadha U., Selvaraj S.K., Thanu S.V., Cholapadath V., Abraham A.M., Zaiyan M., Manoharan M., Paramsivam V. (2022). A review of the function of using carbon nanomaterials in membrane filtration for contaminant removal from wastewater. Mater. Res. Express.

[2] Palani G., Arputhalatha A., Kannan K., Lakkaboyana S.K., Hanafiah M.M., Kumar V., Marella R.K. (2021). Current Trends in the Application of Nanomaterials for the Removal of Pollutants from Industrial Wastewater Treatment—A Review. Molecules.

[3] Saranya R., Arthanareeswaran G., Dionysiou D.D. (2014). Treatment of paper mill effluent using Polyethersulfone/functionalisedmultiwalled carbon nanotubes based nanocomposite membranes. Chem. Eng. J..

[4] Yang Z., Zhou Y., Feng Z., Rui X., Zhang T., Zhang Z. (2019). A review on reverse osmosis and nanofiltration membranes for water purification. Polymers.

[5] Rahul Krishna B., Bhuvaneshwari S., Majeed F., Manoj M.M., Jose E., Mohan A. (2022). Different treatment methodologies and reactors employed for dairy effluent treatment—A review. J. Water Process Eng..

[6] Ji J., Liu F., Hashim N.A., Abed M.M., Li K. (2015). Poly (vinylidene fluoride)(PVDF) membranes for fluid separation. React. Funct. Polym..

[7] Sobola D., Kaspar P., Částková K., Dallaev R., Papež N., Sedlák P., Trčka T., Orudzhev F., Kaštyl J., Weiser A. (2021). PVDF fibers modification by nitrate salts doping. Polymers.

[8] Kaspar P., Sobola D., Částková K., Dallaev R., Šťastná E., Sedlák P., Knápek A., Trčka T., Holcman V. (2021). Case study of polyvinylidene fluoride doping by carbon nanotubes. Materials.

[9] Chang Y.R., Lee Y.J., Lee D.J. (2019). Membrane fouling during water or wastewater treatments: Current research updated. J. Taiwan Inst. Chem. Eng..

[10] Leong S., Razmjou A., Wang K., Hapgood K., Zhang X., Wang H. (2014). TiO_2_ based photocatalytic membranes: A review. J. Membr. Sci..

[11] Akhavan O. (2009). Journal of Colloid and Interface Science Lasting antibacterial activities of Ag–TiO_2_/Ag/a-TiO two nanocomposite thin-film photocatalysts under solar light irradiation. J. Colloid Interface Sci..

[12] Farahani MH D.A., Vatanpour V. (2018). A comprehensive study on the performance and antifouling enhancement of the PVDF mixed matrix membranes by embedding different nanoparticles: Clay, functionalized carbon nanotube, SiO_2_ and TiO_2_. Sep. Purif. Technol..

[13] Zouzelka R., Kusumawati Y., Remzova M., Rathousky J., Pauporté T. (2016). Photocatalytic activity of porous multiwalled carbon nanotube-TiO_2_ composite layers for pollutant degradation. J. Hazard. Mater..

[14] Sisay E.J., László Z. (2021). Trend and Novel Possibilities of Dairy Wastewater Treatment by Membrane Filtration. J. Eng. Sci. Technol. Rev..

[15] Malathi A., Arunachalam P., Kirankumar V.S., Madhavan J., Al-Mayouf A.M. (2018). An efficient visible light driven bismuth ferrite incorporated bismuth oxyiodide (BiFeO_3_/BiOI) composite photocatalytic material for degradation of pollutants. Opt. Mater..

[16] Ratova M., Redfern J., Verran J., Kelly P.J. (2018). Highly efficient photocatalytic bismuth oxide coatings and their antimicrobial properties under visible light irradiation. Appl. Catal. B Environ..

[17] Orimolade B.O., Arotiba O.A. (2020). Bismuth vanadate in photoelectrocatalytic water treatment systems for the degradation of organics: A review on recent trends. J. Electroanal. Chem..

[18] Jung J.-Y., Lee D., Lee Y.-S. (2015). CNT-embedded hollow TiO_2_ nanofibers with high adsorption and photocatalytic activity under UV irradiation. J. Alloys Compd..

[19] Cao Q., Yu Q., Connell D.W., Yu G. (2013). Titania/carbon nanotube composite (TiO_2_/CNT) and its application for removal of organic pollutants. Clean Technol. Environ. Policy.

[20] Coelho F.E.B., Deemter D., Candelario V.M., Boffa V., Malato S., Magnacca G. (2021). Development of a photocatalytic zirconia-titania ultrafiltration membrane with antifouling and self-cleaning properties. J. Environ. Chem. Eng..

[21] Riaz S., Park S.J. (2020). An overview of TiO_2_-based photocatalytic membrane reactors for water and wastewater treatments. J. Ind. Eng. Chem..

[22] Sisay E.J., Veréb G., Pap Z., Gyulavári T., Ágoston Á., Kopniczky J., Hodúr C., Arthanareeswaran G., Arumugam G.K.S., László Z. (2022). Visible-light-driven photocatalytic PVDF-TiO_2_/CNT/BiVO_4_ hybrid nanocomposite ultrafiltration membrane for dairy wastewater treatment. Chemosphere.

[23] Kusworo T.D., Ariyanti N., Utomo D.P. (2020). Effect of nano-TiO_2_ loading in polysulfone membranes on the removal of pollutant following natural-rubber wastewater treatment. J. Water Process Eng..

[24] Kovács I., Veréb G., Kertész S., Hodúr C., László Z. (2018). Fouling mitigation and cleanability of TiO_2_ photocatalyst-modified PVDF membranes during ultrafiltration of model oily wastewater with different salt contents. Environ. Sci. Pollut. Res..

[25] Sisay E.J., Bagi K., Fazekas Á., Kertész Sz Veréb G., László Z. (2020). Filtration of proteins through TiO_2_ photocatalyst modified PVDF membrnaes. Desalination Water Treat..

[26] Nascimben Santos E., Agoston A., Kertész S., Hodúr C., László Z., Pap Z., Veréb G. (2020). Investigation of the applicability of TiO_2_, BiVO_4_, and WO_3_ nanomaterials for advanced photocatalytic membranes used for oil-in-water emulsion separation. Asia-Pac. J. Chem. Eng..

[27] Srivastava H.P., Arthanareeswaran G., Anantharaman N., Starov V.M. (2011). Performance of modified poly(vinylidene fluoride) membrane for textile wastewater ultrafiltration. Desalination.

[28] Ding Y., Ma B., Liu H., Qu J. (2019). Effects of protein properties on ultrafiltration membrane fouling performance in water treatment. J. Environ. Sci..

[29] Lawrence N.D., Perera J.M., Iyer M., Hickey M.W., Stevens G.W. (2006). The use of streaming potential measurements to study the fouling and cleaning of ultrafiltration membranes. Sep. Purif. Technol..

[30] Vatanpour V., Yekavalangi M.E., Safarpour M. (2016). Preparation and characterization of nanocomposite PVDF ultrafiltration membrane embedded with nanoporous SAPO-34 to improve permeability and antifouling performance. Sep. Purif. Technol..

[31] Zhao X., Lan Y., Yang K., Wang R., Cheng L., Gao C. (2020). Antifouling modification of PVDF membranes via in situ mixed-charge copolymerization and TiO_2_ mineralization. Appl. Surf. Sci..

[32] Wang X.L., Qu Z.G., Lai T., Ren G.F., Wang W.K. (2022). Enhancing water transport performance of gas diffusion layers through coupling manipulation of pore structure and hydrophobicity. J. Power Sources.

[33] Wang X.L., Qu Z.G., Ren G.F. (2023). Collective enhancement in hydrophobicity and electrical conductivity of gas dissfusion layer and the electrochemical performance of PEMFCs. J. Power Sources.

[34] Dhand V., Hong S.K., Li L., Kim J.M., Kim S.H., Rhee K.Y., Lee H.W. (2019). Fabrication of robust, ultrathin and light weight, hydrophilic, PVDF-CNT membrane composite for salt rejection. Compos. Part B Eng..

[35] Ismail N., Lau W.J., Ismail A.F., Goh P. (2013). Preparation and Characterization of Polysulfone/Polyphenylsulfone/Titanium Dioxide Composite Ultrafiltration Membranes for Palm Oil Mill Effluent Treatment. J. Teknol..

[36] Selvaraj M., Hai A., Banat F., Haija M.A. (2020). Application and prospects of carbon nanostructured materials in water treatment: A review. J. Water Process Eng..

[37] Liu C., Wu L., Zhang C., Chen W., Luo S. (2018). Surface hydrophilic modification of PVDF membranes by trace amounts of tannin and polyethyleneimine. Appl. Surf. Sci..

[38] Yang C., Wang P., Li J., Wang Q., Xu P., You S., Zheng Q., Zhang Q. (2021). Photocatalytic PVDF ultrafiltration membrane blended with visible-light responsive Fe(III)-TiO_2_ catalyst: Degradation kinetics, catalytic performance and reusability. Chem. Eng. J..

[39] Moghadam M.T., Lesage G., Mohammadi T., Mericq J.-P., Mendret J., Heran M., Faur C., Brosillon S., Hemmati M., Naeimpoor F. (2015). Improved antifouling properties of TiO_2_/PVDF nanocomposite membranes in UV-coupled ultrafiltration. J. Appl. Polym. Sci..

[40] Xie W., Li J., Sun F., Dong W., Dong Z. (2021). Strategy study of critical flux/threshold flux on alleviating protein fouling of PVDF-TiO_2_ modified membrane. J. Environ. Chem. Eng..

