# Supplementary material for: Investigation of Photocatalytic PVDF Membranes Containing Inorganic Nanoparticles for Model Dairy Wastewater Treatment
Source: Membranes (Basel). 2023 Jul 10;13(7):656. doi: 10.3390/membranes13070656 (PMC10383713; doi:10.3390/membranes13070656)
Supplement: Supplementary file 1 [file membranes-13-00656-s001.zip › membranes-2471672-supplementary.pdf]

Supplementary material

Investigation of photocatalytic PVDF membranes containing inorganic nanoparticles for model dairy wastewater treatment

Elias Jigar Sisay [1,2], Ákos Fazekas [1,2,] Tamás Gyulavári[3], Judit Kopniczky[4], Béla Hopp[4], Gábor Veréb[2] and Zsuzsanna László [2,*]

Supplementary material

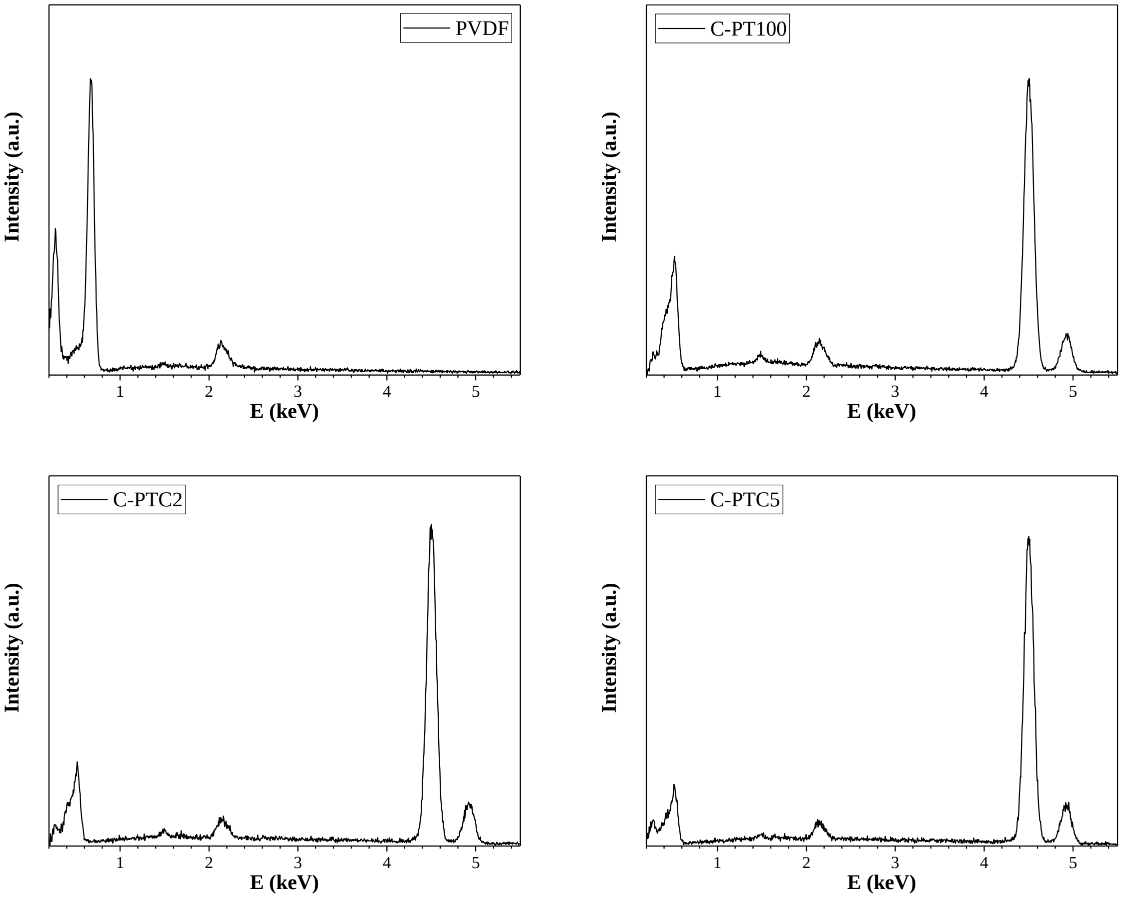

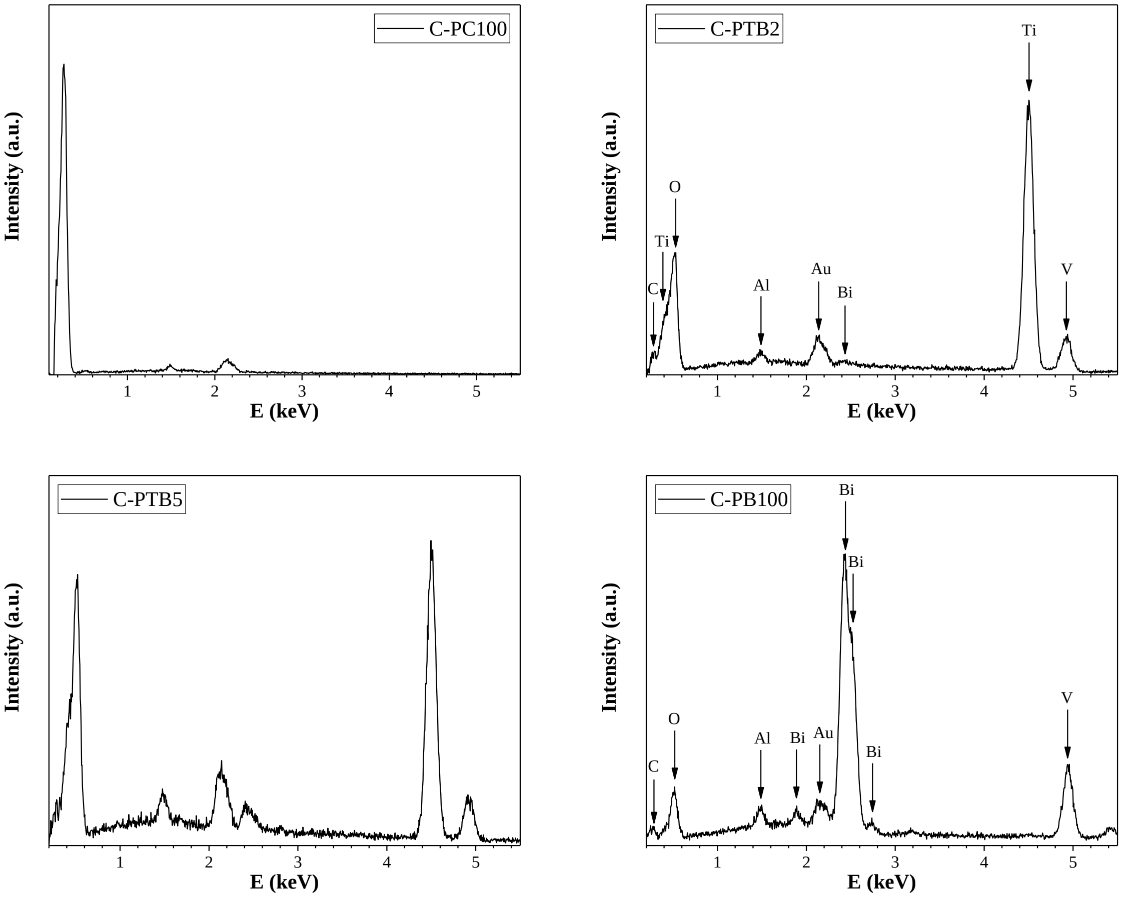

Figure S1. EDX spectra of covered nanocomposite membranes

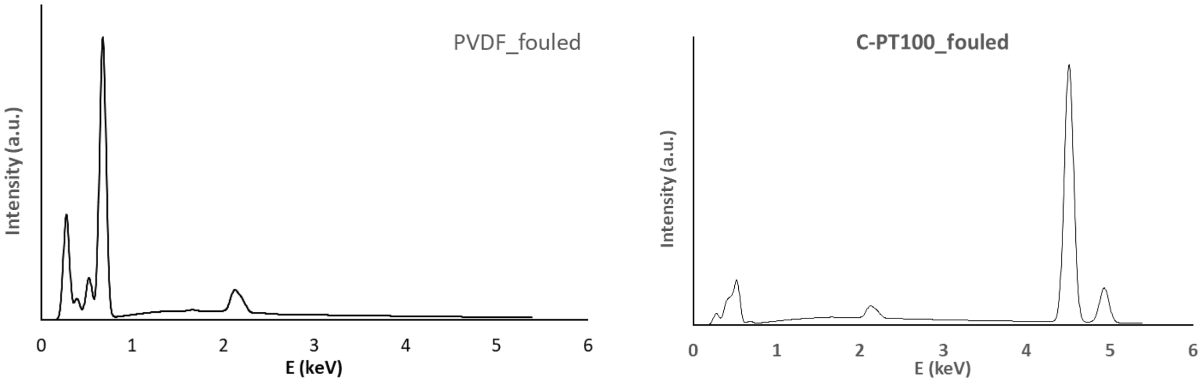

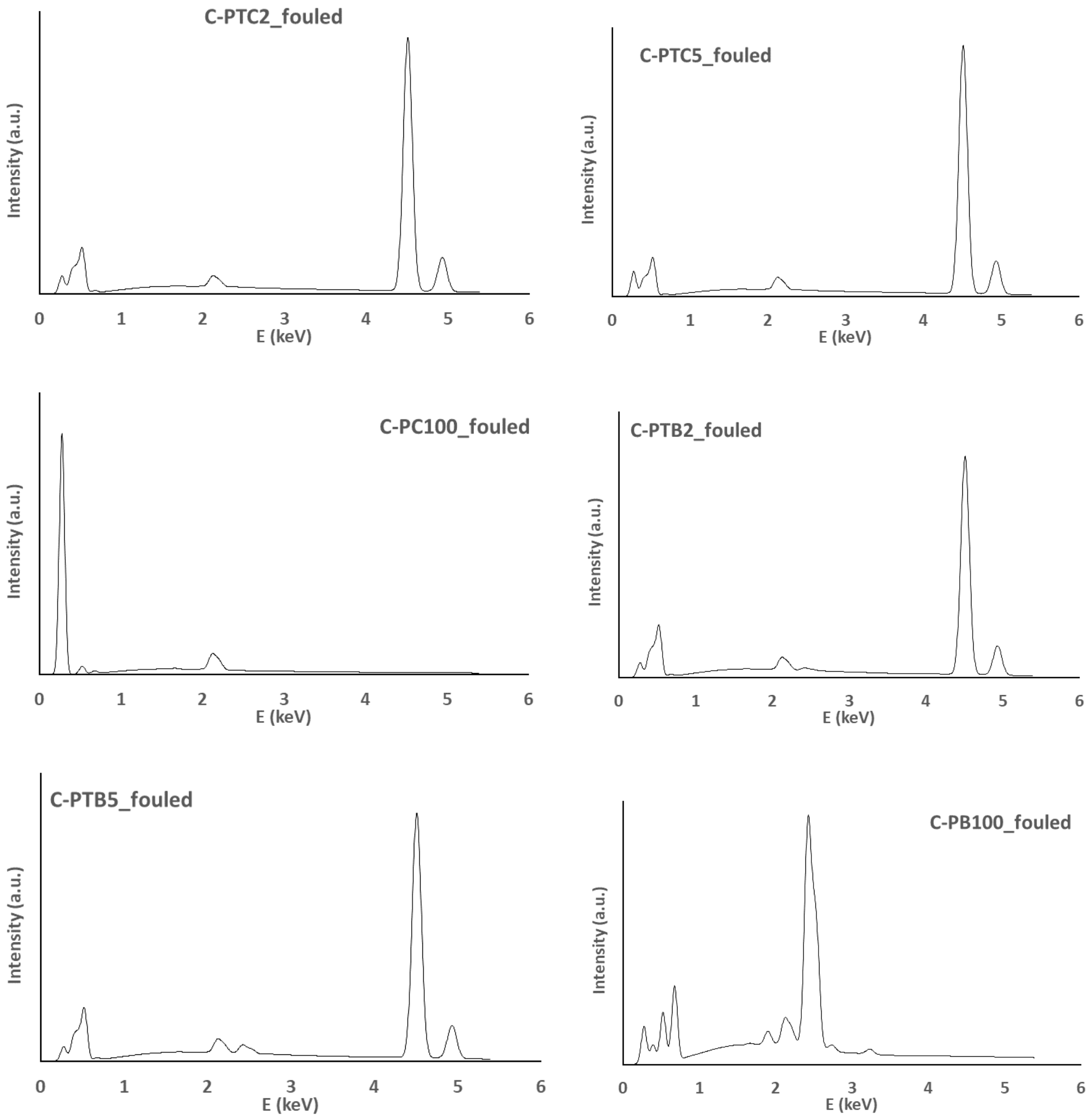

Figure S2. EDX spectra of covered nanocomposite membranes after BSA filtration
